# Decoding Immune Heterogeneity of Melanoma and identifying immune-prognostic hub genes

**DOI:** 10.7150/jca.50277

**Published:** 2021-01-01

**Authors:** Yu Zhang, Siyu Hao, Yingli Gao, Weina Sun, Yuzhen Li

**Affiliations:** Department of Dermatology, the Second Affiliated Hospital of Harbin Medical University, No.248 Xuefu Road, Nangang District, Harbin. 150081, P. R. China.

**Keywords:** melanoma, tumor microenvironment, immune heterogeneity, prognostic biomarkers

## Abstract

Melanoma is an aggressive skin cancer that has gained attention worldwide. Growing evidence has highlighted that the tumor microenvironment (TME) is an important feature of carcinogenesis and contributes to therapeutic efficacy in melanoma. However, additional advances in melanoma immuno-oncology are necessary to achieve a comprehensive knowledge of the immune infiltrate population and to identify accurate and readily measurable biomarkers. In this study, we analyzed gene expression of 468 melanoma cases from the TCGA database, which led to the identification of three melanoma clusters (representedby low, median and high infiltration) that display unique immune features. We found that the microenvironment clusters had substantial prognostic efficacy. The median cluster was characterized by an inability to draw immune cells, highlighting possible immune escape mechanisms, and lower CXCL9 and CXCL10 expression, which was correlated to poor prognosis. Deep molecular characterization of immune cells, cytolytic-activity and tumor-inflammatory status revealed diversity of the local immune infiltration landscape in the melanoma clusters. Differentially expressed genes related to TME were extracted from each infiltration cluster. Functional annotations revealed that these genes were mainly related to immune system activation and the processes of immunoreaction. The top ten hub genes in immune infiltration-related protein-protein interaction (PPI) networks were selected for further prognostic investigation. Further validation showed that five of ten hub genes were good prognostic biomarkers for melanoma in two independent groups from the Gene Expression Omnibus database. In brief, these data highlight that systemic characterization of melanoma could uncover tumor infiltrate characteristics, which can help select the most adequate treatment and identify consistent and important indicators of the local immune tumor microenvironment in melanoma patients.

## Introduction

Melanoma is an aggressive cancer that accounts for more than 160,000 new cancer cases and more than 80% of skin cancer-related deaths worldwide every year 1, 2. Although significant advances in metastatic melanoma treatments have demonstrated some level of success in the last several years, melanoma patients with metastatic lesions still exhibit poor prognosis with a five-year overall survival that ranges from 45% for stage III to 18% for stage IV 3. Over the last decade, tumor microenvironment (TME) studies have already led to variations in treatment methods and have raised hopes for improved treatment of melanoma patients, particularly for patients in the advanced stages 4. Previous studies have shown that increased density of tumour-infiltrating lymphocytes (TILs) are associated with better patient prognosis, as well as a reduced occurrence of lymph node metastasis and a lengthier disease-free survival (DFS) 5, 6. An innovative study by Thomas *et al.* of thousands of melanoma patients suggested that the degree of lymphocyte infiltration was an independent prognosticator of DFS, such that a lesser grade was associated with a reduced DFS 7. These studies implied that assessing the diversity of the TME and transforming the immune microenvironment can be promising strategy for melanoma therapy.

Immunotherapy was considered to be the foundation in melanoma treatment, and is proposed to regulate host immunity against the tumor 8. Immunotherapeutic strategies have demonstrated success and enhanced patient survival 9. During 2011-2014, up to seven new monoclonal antibodies for metastatic melanoma became FDA approved, such as Pembrolizumab (anti-PD1), Nivolumab (anti-PD-1) and Ipilimumab (anti-CTLA4) 4. Despite the fact that immune checkpoint blockade is transforming treatment for advanced cancer, many patients that are being administered these therapies don't demonstrate a long-lasting response and have poor prognosis 10, 11. Due to the complexity of the genomic landscape of tumor samples and the heterogeneity of the TME, it is imperative to characterize the TME in melanoma and to detect biomarkers capable of identifying patients with better prognosis after these treatments. Several studies have used these approaches to explore immune infiltrate characteristics of melanoma. Reuben et al. assess the relevance and relationship of genomic and immune heterogeneity to therapeutic responses 12. Significant heterogeneity of targeted therapy as well as genomic and immune heterogeneity of patients was observed 12. Wolf Y et al. explore the effects of intra-tumor heterogeneity on tumor aggressiveness and immunity using a mouse melanoma model 13. However, a comprehensive landscape of the connections among melanoma and immune cells remains, and TME-related prognostic markers, are still not well-characterized.

In this study, we estimated the immune infiltration pattern of melanoma samples and computed heterogeneity across 428 patients from the TCGA database. These melanoma patients were grouped into three immunophenotypes according to the immune infiltration pattern using unsupervised clustering analysis. Further molecular characterization of immune cells cytolytic-activity and tumor-inflammation revealed diversity in the local immune infiltrate population in the different clusters. Finally, we identified potential immune related genes signatures that are significantly correlated with the patients' prognosis.

## Materials and Methods

### Skin Cutaneous Melanoma (SKCM) datasets and preprocessing

Publicly available SKCM gene-expression data sets with full clinical annotations were obtained from the TCGA database. Transcriptome raw counts of the TCGA-SKCM project were downloaded from GDC (https://portal.gdc.cancer.gov). For the project, the amount of samples, baseline data, and clinical endpoints of every appropriate GDC data set was assessed using R and R Bioconductor packages. Patients that didn't have survival data were not included in further evaluation. Overall, 468 SKCM samples that had transcriptome profiling and clinical features available were included in this study. Also, two independent test datasets (GSE22155 and GES54467) were included. The detailed clinic parameters of enrolled patients were shown in Table S1.

### Calculation of microenvironment cell abundance

The deconvolution algorithm CIBERSORT 14 was employed to calculate the proportion of immune cells in SKCM samples. The LM22 gene signature was used, which included specific markers of 22 human immune cell types, such as T-cells, B-cells, natural killer cells, DCs, macrophages and myeloid cells. CIBERSORT utilizes a database of reference gene-expression values, composed of 547 genes (a minimal representation for cell types). The data was used to infer proportion of cell types from bulk tumor samples with assorted cell types through the use of support vector regression. The algorithm with the LM22 sets and 1,000 permutations estimated the percentage of stromal cells by instituting the Microenvironment Cell Populations-counter method. This permits vigorous computation of the absolute number of eight immune and two stromal populations in diverse tissues from transcriptomic information 15.

### Identification of immune subsets

Unsupervised hierarchical agglomerative clustering was used to group tumor samples with qualitatively similar tumor immune cell infiltration patterns into immune subtypes (established using the Euclidean distance and Ward's linkage). We utilized clustering results to reorganize samples and scaled the CIBERSORT data prior to heatmap plotting (“pheatmap” function in R).

To designate the relevant pattern (i.e. relative cell proportion) of immune cell subsets in three clusters, the tumor purity, immune score and stromal score were also calculated using “estimate” package 16.

### Characterization of Immune Gene Signatures among the SKCM Immune clusters

Cytolytic activity (CYT) was computed using an already validated gene expression signature using the geometric average of granzyme A (GZMA) and perforin-1 (PRF1) gene expression 17. Tumor Inflammation Signature (TIS) was quantified using the average of continuous mean of log2-transformed normalized expression of known genes 18. Relative antigen presentation machinery (APM) was quantified using a validated gene expression signature 19. Pathological assessment of the proportion of tumor infiltrating lymphocytes (TILs) 20 was also used.

### Prognostic analysis of immune clusters

Both the univariate and multivariate Cox proportional hazard model was created to assess the predictive importance of immune clusters. Age, tumor grade and gender were first examined using the univariate Cox proportional hazards model. All factors of significance were encompassed as covariates in the multivariate Cox proportional hazards model. Survival outcomes modeled data in relation to disease-free survival (DFS). In particular, events were described as death according to any cause, with the time being precisely to the day. *P*-values were acquired from univariate Cox proportional-hazards regression models through the R package survival. All Kaplan-Meier survival curves were visualized through the use of the survfit function in the computational survival package.

### Differentially expressed genes (DEGs) between the tumor immune clusters

To determine genes that are associated with different infiltrating patterns, DEGs amongst groups were identified using the R package limma 21. Due to the significant survival difference and small sample size of low infiltration subtype, DEGs were calculated between high infiltration cluster and median cluster using the criteria of adjusted *P* value < 0.05 and logFC>1. The adjusted *P*-value for multiple testing was quantified utilizing the Benjamini-Hochberg correction.

### Enrichment analysis

Genes that were differentially expressed between high and median subtypes were identified as DEGs and used for functional enrichment analyses. The analyses were performed using the online gene annotation and analysis tool Metascape (http://metascape.org) 22. Search Tool for the Retrieval of Interacting Genes (STRING, http://string-db.org) online database was utilized for prediction of PPI network of significant DEGs (combined score >0.9). Cytoscape 3.7.2 was utilized to visualize interactive network.

### Statistical analysis

Statistical analysis was conducted through utilization of R version 3.5.0. Distribution of inflammatory markers within immune clusters were visualized using box plots and differences in median values were evaluated by ANOVA followed by post-hoc testing (*p-value < 0.05; **p-value < 0.005; ***p-value < 0.0005; ****p-value < 0.00005). Unadjusted (crude) and age-adjusted odds ratios (ORs), with 95% confidence intervals (CIs), were quantified. Unsupervised clustering for tumor samples and immune cell types was conducted using hierarchical clustering. Correlation between immune factors was calculated using Pearson's correlation coefficients. Survival analysis was conducted through the Kaplan-Meier technique. The survival of each cluster was compared through use of the log rank test.

## Results

### Microenvironment phenotypes in SKCM

**Figure 1:**
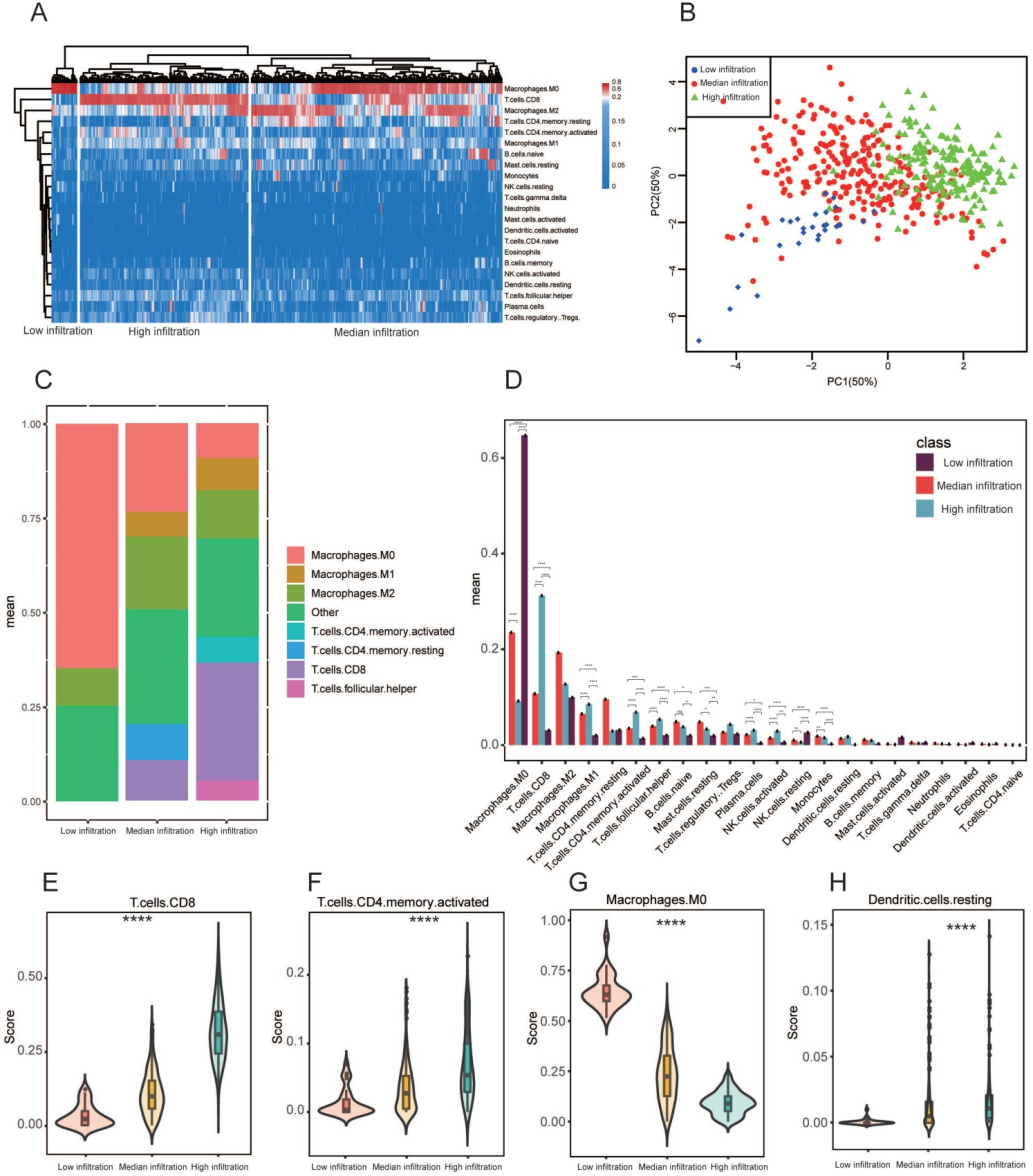
** Microenvironment phenotypes in melanoma. (A)** Hierarchical agglomerative clustering of melanoma microenvironment phenotypes established using predicted numbers of 22 microenvironment cell subsets computed using CIBERSORT.** (B)** Principal component analysis (PCA) of the immune clusters computed as a linear mixture of immune-related genes. Component 1 and 2 account for 100% of the variation. **(C)** Relative proportion of immune cell populations across each cluster. Bars demonstrate the average proportion of 7 aggregated immune cell populations. Clusters are on the x-axis, and the average composition of immune cells compartment is on the y-axis. Colors are exhibited by individual immune cell proportions. **(D)** Bars display the average proportion of 22 immune cell populations. Immune cell population is located on the x-axis while the y-axis shows the average proportion of the immune cell component, including the SEM. All cluster subtypes are incorporated. **(E-H)** Signature scores of CD8+ T cells, resting dendritic cells, CD4+ memory activated T-cells and M0 macrophages among clusters. The boxplot is located within the violin plot. * *P* < 0.05; ** *P* < 0.005; *** *P* < 0.0005; **** *P* < 0.00005.

Heterogenous immune cell populations penetrate the tumor microenvironment and regulate the anti-tumor response. To assess the range of immune cell infiltration, 468 SKCM patients with available transcriptome data and clinical features were incorporated in our analyses. Using CIBERSORT results, we performed hierarchical agglomerative clustering of the 22 human immune cell phenotypes. All 468 patients were categorized into three heterogeneous clusters (cluster 1=264, cluster 2=177, cluster 3=27). A significant heterogeneity was observed with regards to infiltration of several immune cell types in the cohorts (Figure 1A). According to level of CD8+ T cell infiltration, the 468 samples were classified into 3 immune clusters (low infiltration (27), median infiltration (264) and high infiltration (177); Figure 1A). Principal component analysis (PCA) exhibited vigorous variations in profiles among the three clusters detected using the hierarchical agglomerative clustering (Figure 1B). In three immune clusters, the aggregated macrophage (M0, and M2 populations) and CD8+ T cells population comprised the largest percentage of cell compartment (Figure 1C). After correcting for multiple comparisons, the average proportion of M0 macrophages, M1 macrophages, CD8+ T cells, and CD4+ memory activated T cells, monocytes and plasma cells were significantly difference between the three heterogeneous clusters (*P* adjusted < 0.005 respectively) (Figure 1D). We additionally assessed additional immune signatures to corroborate our immune clustering. The distribution of CD8+ T cells, resting dendritic cells, CD4+ memory activated T cells and M0 macrophages also coincided with our established microenvironment clusters (Figure 1E-H). Meanwhile, the ESTIMATE algorithm was employed to evaluate tumor purity, stromal and immune scores within the immune clusters and their relationships. We discovered significant variation in immune scores, stromal scores and tumour purity among the three immune clusters (Figure S1 A-C). We also observed significant association of stromal and immune scores with tumour purity predictions (Pearson's correlation coefficient: -0.86 and -0.93) (Figure S1D, E). Importantly, we found a positive correlation between stromal and immune scores (Pearson's correlation coefficient: 0.74). ESTIMATE scores demonstrated a high correlation with tumor purity in comparison with stromal and immune scores (Figure S1G).

### Prognostic significance of immune clusters in SKCM

**Figure 2:**
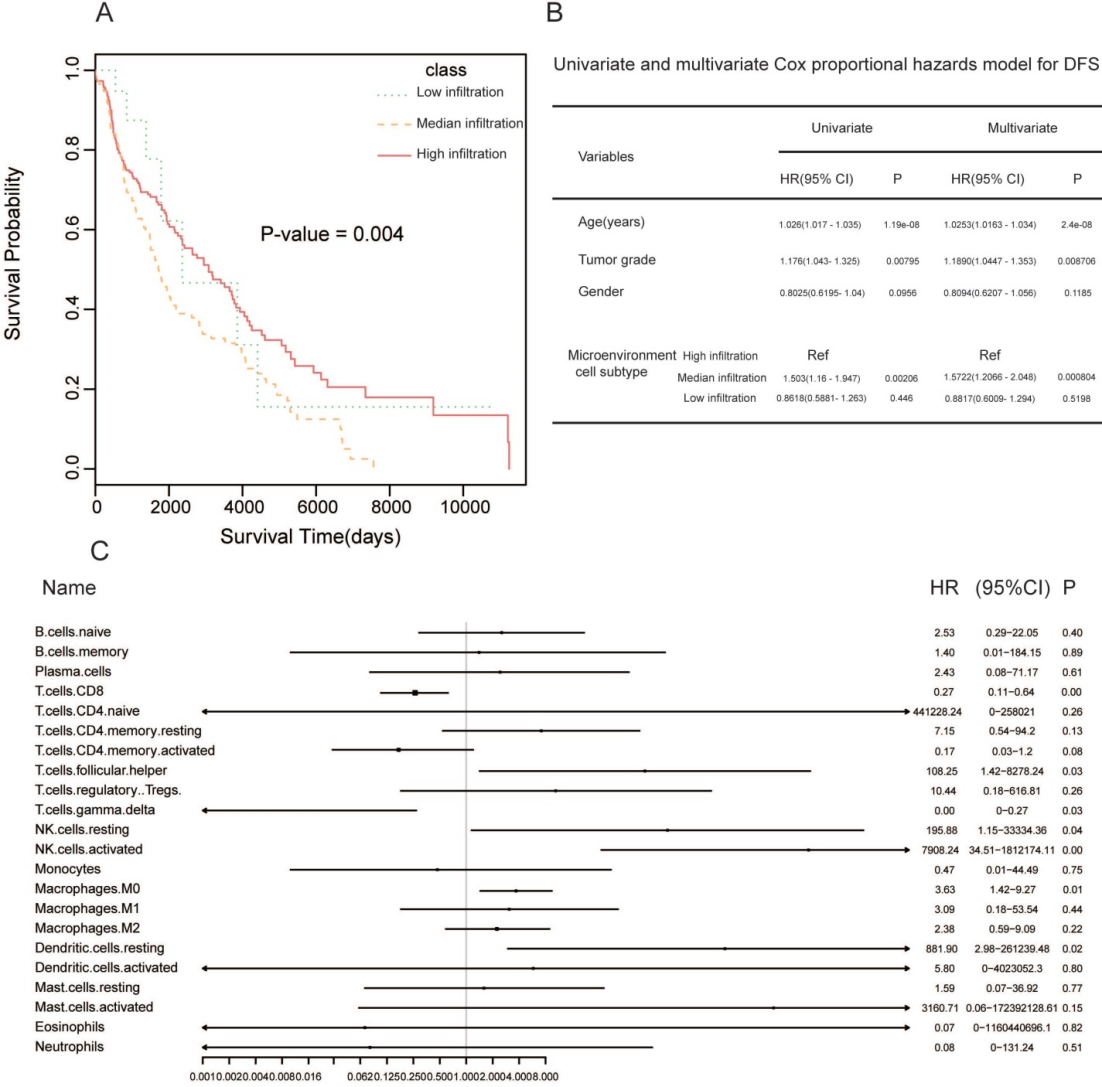
** Prognosis of immune cells in melanoma. (A)** Kaplan-Meier curves for overall survival (OS) of 468 melanoma patients with the tumor microenvironment infiltration classes with log-rank test score. **(B)** Hazard ratios (HRs) and P values of the covariates in the univariate and multivariate Cox proportional hazard model for DFS. **(C)** Subgroup analyses approximating clinical prognostic value among CIBERSORT result cohorts in independent melanoma data. Horizontal line length exhibits 95% confidence interval for each cell populations. The hazard ratio (HR) is represented by the vertical dotted lines. The vertical solid line represents HR = 1. HR < 1.0 indicates if a cell population has a favorable prognostic biomarker.

To investigate how tumor microenvironment affects prognosis, we determined the clinical significance of microenvironment clusters. Hierarchical clustering showed considerable differences in survival between the three clusters (log rank *P* = 0.004). High infiltration cluster had significantly better overall survival (OS) compared to the additional two clusters (Figure 2A). The multivariate Cox proportional hazards model showed that the high infiltration cluster independently forecasted improved OS in SKCM (HR = 1.5722, 95% CI: 1.2066-2.048, *P* = 0.000804, Figure 2B). Next, we conducted univariate Cox regression to identify whether the 22 human immune cell phenotypes affected patient outcomes. As expected, CD8+ T cells and gamma delta T cells were significantly correlated to overall survival, and high levels of these cell types related to better patient prognosis (CD8 T cells: *P* = 0.009, HR = 0.27; gamma delta T cells: *P* = 0.03, HR = 0.00). At the same time, high levels of M0 macrophages were associated with improved patient prognosis (M0 macrophages: *P* = 0.00, HR = 0.27) (Figure 2C).

### Tumor Immune-features between immune subtypes

**Figure 3:**
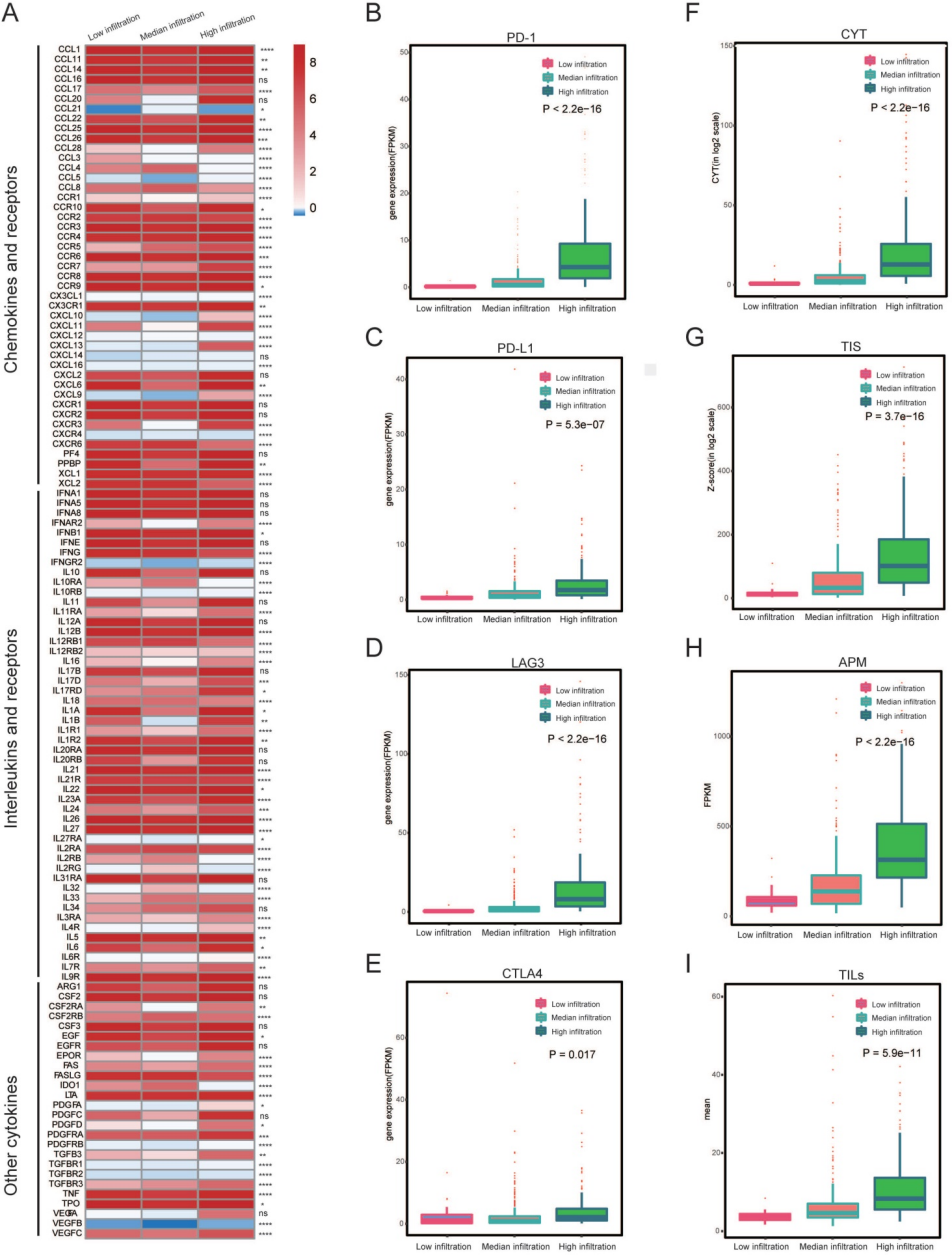
** Heterogeneous immune cell infiltration in melanoma. (A)** Log2-fold change in gene expression of chemokines, ILs, IFNs, and other important cytokines and their receptors for each cluster. Molecules that were differentially expressed among the three heterogeneous clusters (*P* < 0.05) were illustrated. **(B-E)** Expression of PD-1, PD-L1, LAG3 and CTLA4 among immune infiltration clusters by overall immune cell infiltration. **(F)** Comparison of relative cytotoxic activity scores (CYT) between the three heterogeneous clusters of melanomas (*p* < 2.2e-16). **(G)** Relative T-cell infiltration score (TIS) between three heterogeneous clusters of melanomas (*p* = 3.7e-16). **(H)** Relative antigen presentation machinery (APM) between the three heterogeneous cluster of melanomas (*p* < 2.2e-16). **(I)** Pathological evaluation of the percentage of tumor infiltrating lymphocytes (TILs) between three heterogeneous cluster of melanomas (*p* = 5.9e-11).

To determine if different immune subtypes established using gene expression are reminiscent of pathological assessments of tumor infiltrating immune cells, we investigated some tumor immune features (including the expression of interleukins and chemokines (Figure 3A), levels of CYT, TIS as well as the level of checkpoints) among the three immune subtypes. We found that most of the interleukins and chemokines were differentially expressed among the immune subtypes. We also determined the expression of several key immuno-checkpoints (PD-1, PD-L1, LAG3 and CTLA4) in three immune subtypes. These checkpoints were drastically increased in the high infiltration cluster, in comparison to the other two clusters (Figure 3B-E). Then, we found that the high infiltration cluster had the highest level of CYT, which served as a substitute for measuring the degree of antitumor response (*P* < 2.2e-16) (Figure 3D). Meanwhile, significantly higher APM, TILs and TIS were also observed in high infiltration cluster (Figure 3F-I), as well as the IFN-γ (Figure S2). These results indicate that the high infiltration cluster is associated with active immunoediting process. These results were consistent with the expression of chemokines (Figure 3A).

**Figure 4:**
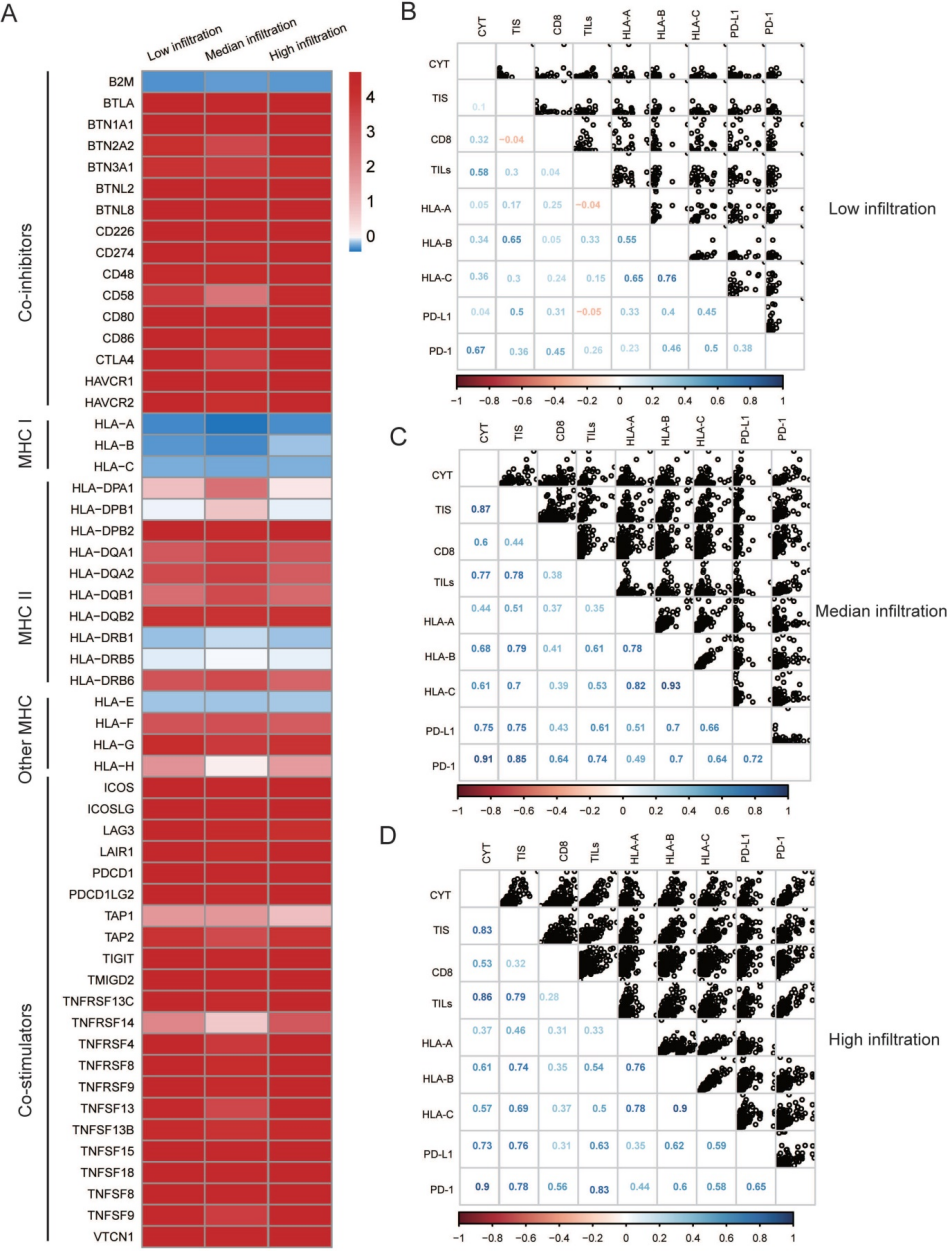
** Possible extrinsic immune escape mechanisms of melanoma. (A)** Log2-fold change in gene expression of chemokines, ILs, IFNs, and other important cytokines and receptors for every cluster. Molecules that are significantly differentially expressed between three heterogeneous clusters (*P* < 0.01) were illustrated. **(B~D)** Correlation matrix of local immune features and MHC-I molecules among three heterogeneous clusters, measured by Spearman R coefficients. CD8+: CIBERSORT result of CD8+ T cells term; TILs: Pathological evaluation of the percentage of tumor infiltrating lymphocytes. Cytolytic activity (CYT) was quantified using a validated gene expression signature established using the geometric mean of gene expression of granzyme A (GZMA) and perforin-1 (PRF1). Pathological assessment of tumor infiltrating lymphocytes (TILs) proportion.

Moreover, both the median and low infiltration clusters exhibited reduced MHC I-related antigen-presenting molecules compared to the high infiltration cluster (Figure 4A, all *P* < 0.05), which is likely attributed to their low immunogenicity. Additionally, we explored the association between immune infiltration (for example, TILs and CYT), immunogenicity, and immune checkpoint molecules. We found that immune infiltration and the majority of checkpoint molecules were positively correlated in the median and high infiltration cluster, whereas this correlation seemed to be diminishing in the high infiltration cluster (Figure 4B-D).

### DEGs screening and functional analysis of immune subtypes

**Figure 5:**
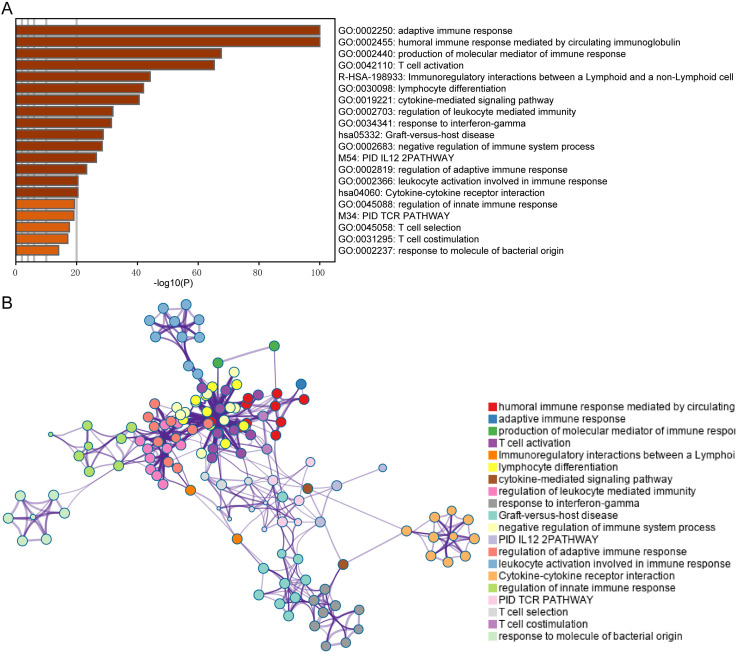
** Functional analysis of the DEGs between immune subtypes. (A)** Top 20 Biological processes correlated to DEGs. **(B)** The network of the top 20 clusters of enriched terms.

In this study, immune-related genes were identified and compared between the high and median infiltration clusters. A total of 813 genes including 16 downregulated and 797 upregulated genes were found in the high infiltration cluster. Furthermore, we conducted GO and pathway enrichment analyses of these genes using Metascape. We established that the genes are involved in the adaptive immune response, lymphocyte differentiation and cytokine-cytokine receptor interaction (Figure 5A and B).

### Identification and validation of hub prognostic genes

**Figure 6:**
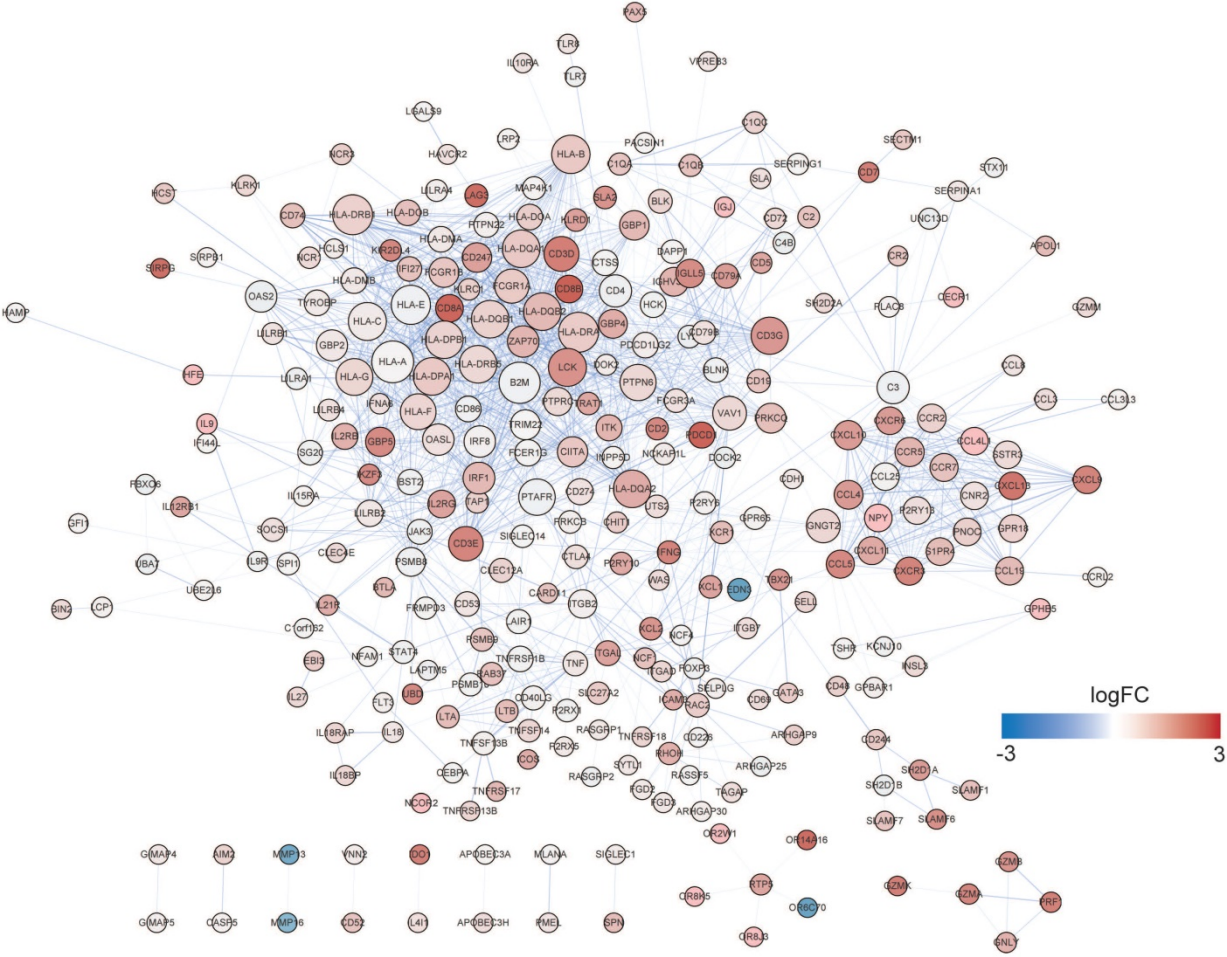
** PPI network of DEGs.** The node size and color represent degree and fold change value.

**Figure 7:**
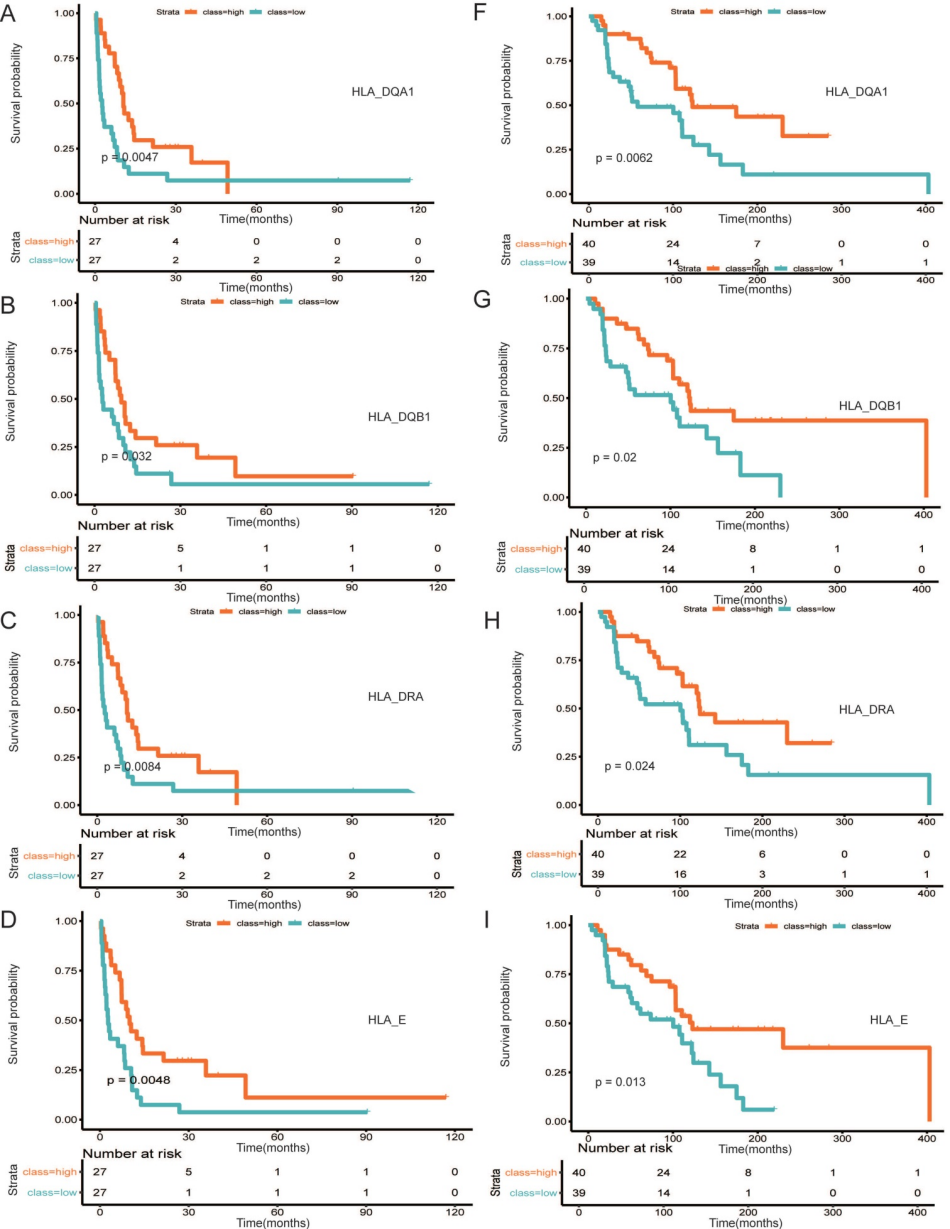

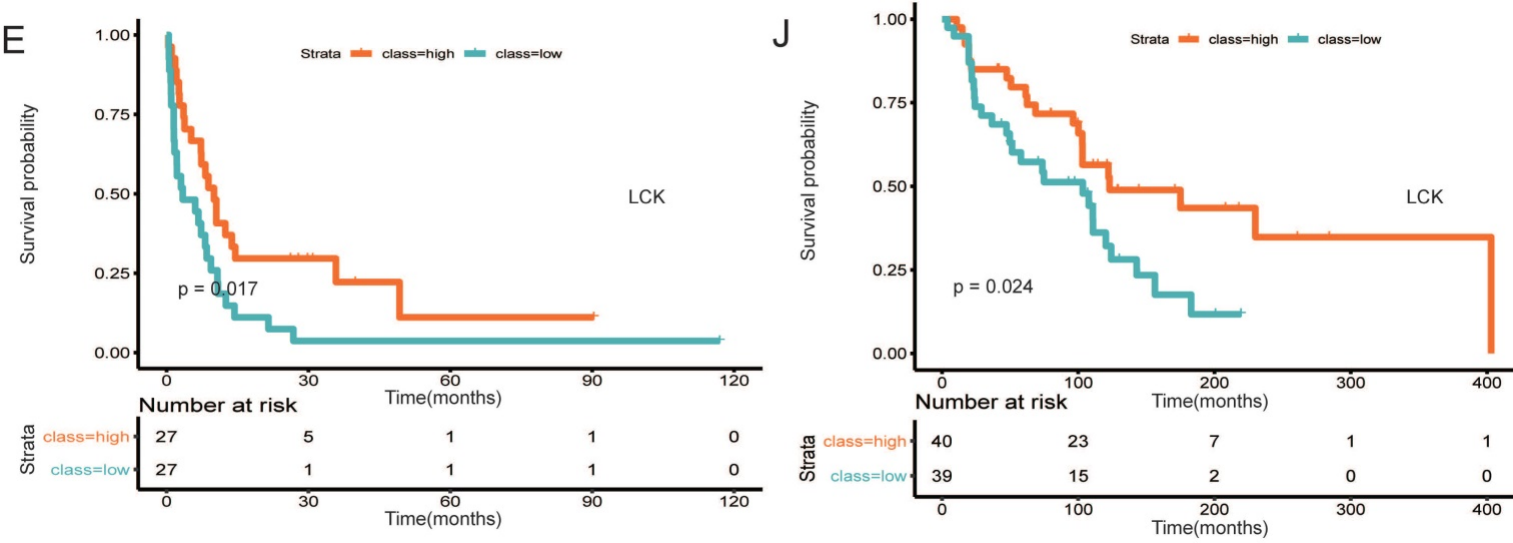
** Survival analysis of five hub (HLA-DQA1, HLA-DQB1, HLA-DRA, HLA-E, LCK) genes in two cohorts. (A-E)** GSE22155, (7F-7J) GES54467.

A PPI network was established using STRING (Figure 6). A total of 291 DEGs connected by 1801 edges were included in the network. The top ten hub genes were selected according to the degree of connectivity. In order to validate the hub genes determined by the TCGA analysis, we used additional melanoma cases obtained from two independent datasets (accession numbers GSE22155 and GES54467). Survival analyses were performed grouped by the expression level of ten hub genes. Table 1 shows the p-value of K-M analysis on the two cohorts. We found that majority of hub genes were significantly correlated to prognosis (Table 1). Increased expression levels of five of the 10 hub genes (HLA-DQA1, HLA-DQB1, HLA-DRA, HLA-E, LCK) were related with improved overall survival of melanoma patients in both validation datasets (Figure 7A-7E: GSE22155; 7F-7J: GES54467).

## Discussion

Intratumor and intertumor heterogeneity of melanoma had been considered as a major obstacle to the successful application of current therapies 23-27. Gaining insights into the cellular and molecular context of melanoma heterogeneity will not only improved the diagnose markers but also promote the evolution of precision medicine. Especially, the studies of TME heterogeneity have raised hopes for improved treatment of melanoma patients 26, 28. The emergence of some computational methods to predict the proportion of immune cells in tumors infiltrates can greatly improve the identification of TME heterogeneity 20, 29. Also, the genomic and transcriptomic sequences of large groups of tumors produced by international projects, including The Cancer Genome Atlas (TCGA, http://cancergenome.nih.gov/), have afforded a chance to determine the characteristic molecular attributes of cancer in unparalleled detail. Despite the fact that several studies have used these approaches to explore immune infiltrate characteristics of melanoma and its association with prognosis 30-32, a comprehensive landscape of the connections among melanoma and immune cells remains unknown.

In this study, we assessed the immune states and diversity of infiltrating populations in melanoma based on TCGA data. The 468 melanoma samples were classified into three immune clusters (low infiltration (n=27), median infiltration (n=264) and high infiltration (n=177). Within the three immune clusters, the combined macrophage population (M0, and M2 populations) and CD8+ T cells population comprised the largest percentage of the cell compartment. Tumor-associated macrophages (TAMs) are the main components of TME 33. Accumulating evidence has demonstrated that TAMs are associated with survival 34, 35. Under differing stimuli, macrophages can be polarized into either the M1 or M2 macrophages. M1 macrophages demonstrate anti-tumor activity 36. Conversely, M2 macrophages have been shown to have pro-tumor activity through promotion of HCC cell proliferation, migration, angiogenesis, and immunosuppressive microenvironment 37, 38. Interestingly, it is has been shown that the level of macrophages (M0 and M2) are inversely proportional to that of CD8+ T cells, suggesting that increased infiltration of CD8+ T cells and low M2 macrophages contributes to anti-tumor immunity.

Considering the importance of the tumor microenvironment with regards to prognosis, we next examined the clinical significance of microenvironment clusters. Median infiltration cluster had worse OS than the other two clusters (*P*-value = 0.004; Figure 2A), indicating that an increased infiltration of immune cells predicted better prognosis. The worse survival associated with median infiltration cluster might be due to the relatively higher infiltration of M2 macrophages, which is reduced in the low infiltration cluster (Figure 1A), and lower expression of some chemokines (Figure 3A). For example, the median cluster has lower expression of CXCL9 and CXCL10, which have been shown to be associated with TIL infiltration in human cancers and draw dendritic cells (DC) and CD8+ T cells 39-41. Then, we investigated immune checkpoints expression, such as CTLA4, PD-1, LAG3, and PD-L1. We found that they were all significantly upregulated in the infiltration cluster (Figure 3B-E). CYT is an important index that delivers a possible way to evaluate the status of the immune microenvironment and immune checkpoints 42. It was established that high levels of CYT value was correlated to enhanced prognosis 43. Our results show that the high infiltration cluster has the highest expression of CYT (Figure. 3F). Meanwhile, APM, TILs, TIS and IFN-γ also show the highest level in the high infiltration cluster but lowest in the low infiltration cluster. (Figure 3F-I; Figure S2). The differences in the characteristics of immune infiltration across the three clusters indicated a distinct intrinsic microenvironment.

In 2009, Camus *et al.* first defined the three major immune coordination profiles (hot, altered and cold) in primary CRCs 44. This immune categorization has been verified in additional cancer types 45. However, there has been a lack of studies on the mechanisms of dysfunctional immune infiltration in melanoma. The hot tumors are characterized by specific features such as the presence of TILs, and the expression of checkpoints 46, 47. We found that these features were present in high infiltration cluster. On the other hand, aside from having poor infiltration, cold tumors have been thought to be immunologically ignorant (a lack of PD- L1 expression) and described by reduced expression of antigen presentation machinery markers including the major histocompatibility complex class I (MHC I) 47, which is consistent with results shown in Figure 4A. These results indicate that the high infiltration cluster in our study might represent a “hot” tumor, whereas the low infiltration cluster represents a “cold” tumor. In addition, we speculated that low expression of MHC I molecules might be responsible for the intrinsic immune escape mechanism poor survival in the median cluster. Interactions amongst the immune system and cancer are governed by a complex interaction of biological pathways. We explored the relationship between immune infiltration factors across the three clusters. We found that immune infiltration and most checkpoint molecules, as well as the CYT, were positively correlated in the high infiltration cluster. Conversely, this was not seen in the low infiltration cluster, suggesting the interactions within this cluster are promiscuous. These results indicated dynamic changes in the immune infiltration stages of melanoma.

We then attempted to explore DEGs of significant prognostic value associated with TME to understand aggressive tumor progression in melanoma patients. A total of 813 DEGs were identified. Functional analysis suggested that they were part of the adaptive immune response, lymphocyte differentiation and cytokine-cytokine receptor connection (Figure 5A and B), which has been revealed to have an important function in activation and modulation of innate immune signaling in the host response to pathogens and cancer 48, 49. In the PPI network, the top 10 hub nodes were extracted and were considered to be an immune-related prognostic marker. At last, upon cross‐validation with two GEO databases composed of data from two independent groups of melanoma patients, we found that five of the 10 hub genes were related to better overall survival of melanoma patients in both validation datasets. These genes were HLA-DQA1, HLA-DQB1, HLA-DRA, HLA-E and LCK. The first four genes are MHC molecules, which have been shown to be related to the progression of melanoma 50, 51. LCK, also known as lymphocyte-specific protein tyrosine kinase, is part of the Src family of non-receptor protein tyrosine kinases 52. These functions have a vital role function in cellular processes such as cell cycle control, cell adhesion, motility, growth, and differentiation 52. Lymphocyte-specific kinase (Lck) is a positive regulator of inflammatory signaling and a druggable target for treatment of cancer and neuronal diseases 53.

In summary, we characterized the TME of melanoma and identified five hub genes that were closely associated with prognosis in melanoma using a series of bioinformatics analyses. Our findings contribute more knowledge of the fundamental molecular mechanisms of how immune molecules and cells affect melanoma progression and therapy. However, additional studies are still necessary to determine the exact mechanism in detail.

## Supplementary Material

Supplementary figures and tables.

## Figures and Tables

**Table 1:** KM analysis *P*-value of hub genes in validated dataset

Name	degree	*P*-value in GSE22155	*P*-value in GSE54467
HLA-A	59	0.0013	0.58
B2M	57	0.082	0.48
HLA-DRB1	54	0.27	0.98
HLA-DRA	54	0.0084	0.024
HLA-E	52	0.0048	0.013
HLA-B	50	0.2	0.16
LCK	49	0.017	0.024
HLA-C	49	0.074	0.32
HLA-DQA1	48	0.0047	0.0062
HLA-DQB1	48	0.032	0.02
