# Supplementary material for: Decoding Immune Heterogeneity of Melanoma and identifying immune-prognostic hub genes
Source: J Cancer. 2021 Jan 1;12(3):703–16. doi: 10.7150/jca.50277 (PMC7778557; doi:10.7150/jca.50277)
Supplement: Supplementary file 1 — Supplementary figures and tables. [file jcav12p0703s1.pdf]

Figure s1

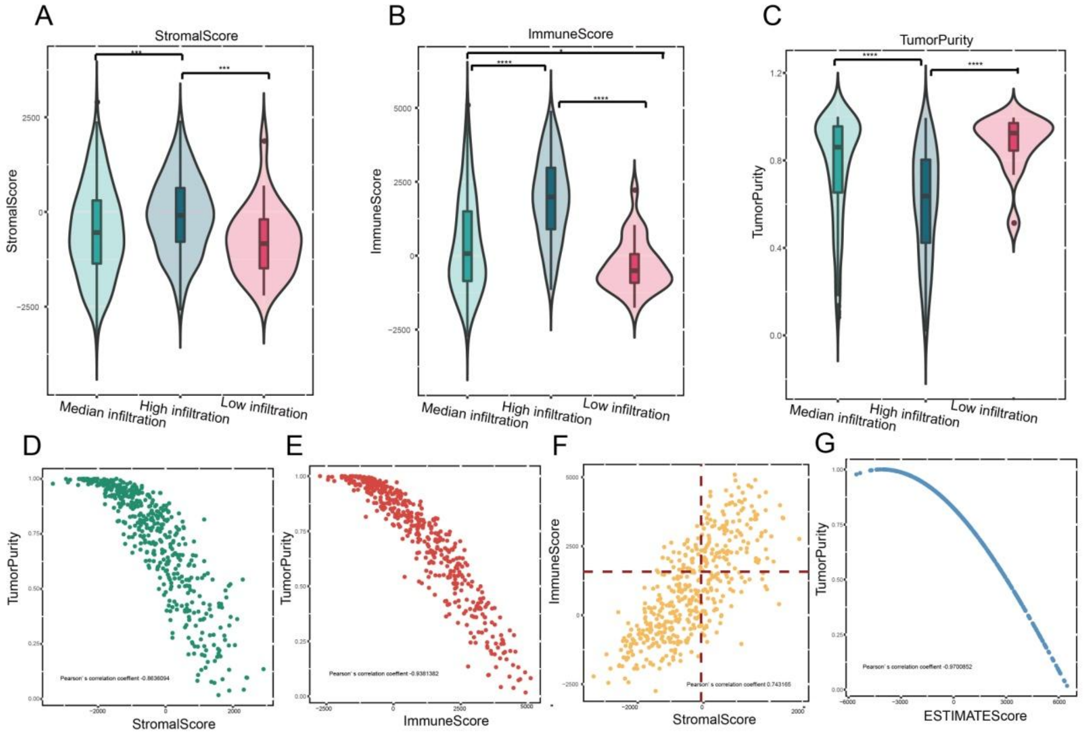

Figure S1. The association between tumour purity variables in the MELANOMA dataset. Comparison of ESTIMATE algorithm results between three heterogeneous cluster. (A) Stromal score. **(B)** Immune score. **(C)** Tumor Purity. (D–G) Scatterplots between tumor purity and (D) stromal, (E) immune, (F) stromal and immune scores and between (G) ESTIMATE scores in MELANOMA dataset**.**

Figure S2

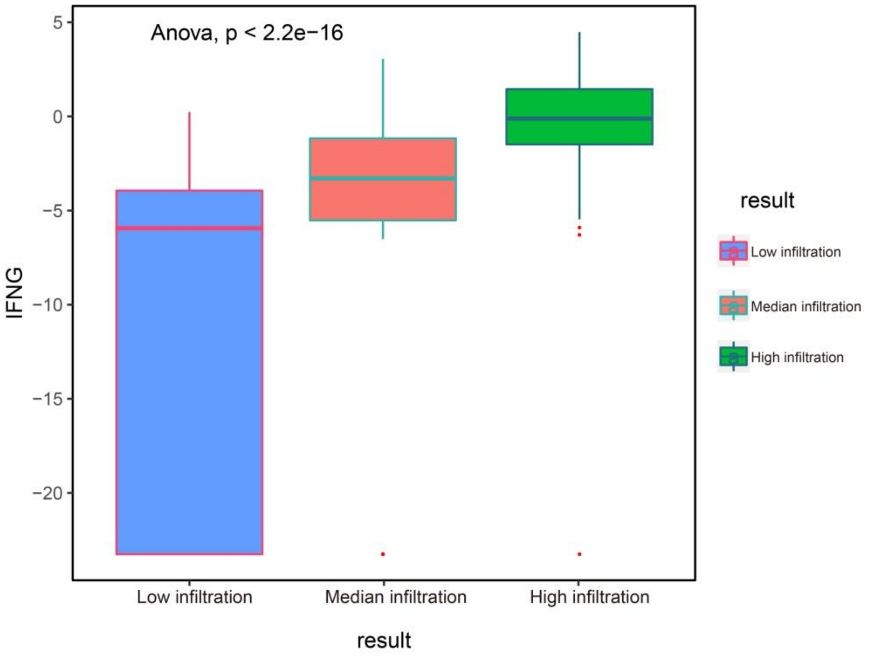

Figure S2.The expression of IFNG among immune infiltration clusters.

Figure s3

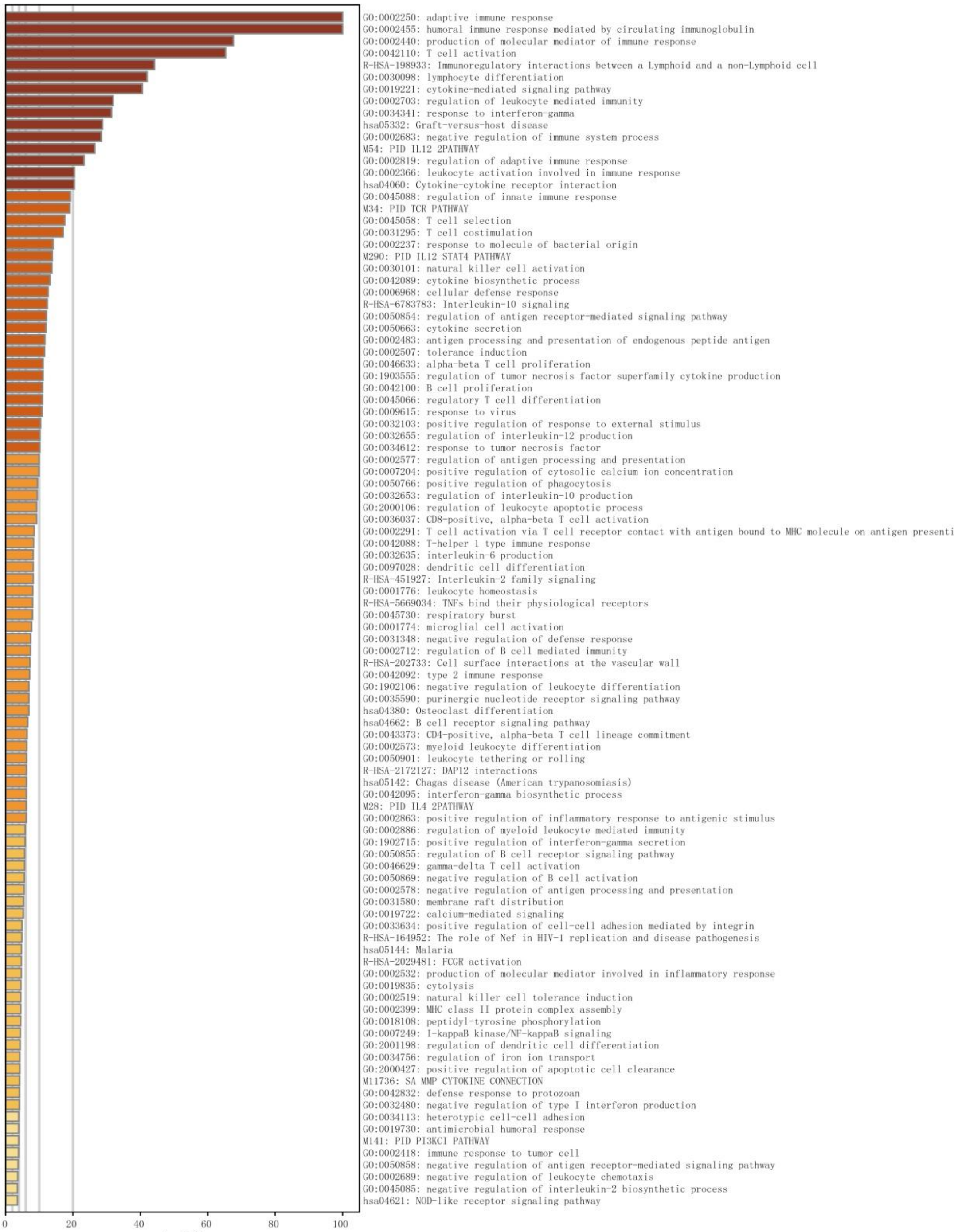

Figure S3. Top 100 Biological processes correlated to the DEGs

Table S1 Clinic information of enrolled patients

	Sample number		
	TCGA-SKCM	GSE22155	GSE54467
All cases	477	57	79
Gender			
Female	181(37.9%)	26(45.6%)	29(36.7%)
Male	296(62.1%)	31(54.4%)	50(63.3%)
Age			
≥ 60	222(46.5%)	26(45.6%)	35(44.3%)
< 60	247(51.8%)	31(54.4%)	44(55.7%)
Type of metastases			
Lymphnode	NA	17(29.8%)	NA
Subcutaneous	NA	38(66.7%)	NA
Lymohnode	NA	1(1.8%)	NA
Type			
NMM	NA	19(33.3%)	NA
SSM	NA	19(33.3%)	NA
Stage			
0	7(1.5%)		
I	78(16.4%)		29(36.7%)
II	140(29.4%)		29(36.7%)
III	175(36.7%)	3(5.3%)	20(25.3%)
IV	24(5.0%)	54(94.7%)	
Number of primary melanomas			
1	NA	NA	69(87.3%)
2	NA	NA	7(8.9%)
3	NA	NA	2(2.5%)
Clark			
I	6(1.3%)	3(5.3%)	NA
II	18(3.8%)	2(3.5%)	NA
III	78(16.4%)	11(19.3%)	NA
IV	168(35.2%)	15(26.3%)	NA
V	55(11.5%)	5(8.8%)	NA
Pathologic_M			
M0	422(88.5%)	NA	NA
M1	25(5.2%)	NA	NA
Pathologic_N			
N0	236(49.5%)	NA	NA
N1	76(15.9%)	NA	NA
N2	50(10.5%)	NA	NA
N3	57(11.9%)	NA	NA
NX	36(7.5%)	NA	NA
Pathologic_T			
T0	23(4.8%)	NA	NA
T1	42(8.8%)	NA	NA
T2	79(16.6%)	NA	NA
T3	92(19.3%)	NA	NA
T4	155(32.5%)	NA	NA
TX	48(10.1%)	NA	NA
